# Risk factors for overweight and obesity, and changes in body mass index of Chinese adults in Shanghai

**DOI:** 10.1186/1471-2458-8-389

**Published:** 2008-11-21

**Authors:** Xuhong Hou, Weiping Jia, Yuqian Bao, Huijuan Lu, Shan Jiang, Yuhua Zuo, Huilin Gu, Kunsan Xiang

**Affiliations:** 1 Department of Endocrinology and Metabolism, Shanghai Jiao Tong University Affiliated Sixth People's Hospital, Shanghai Diabetes Institute, Shanghai Clinical Center of Diabetes, Shanghai 200233, PR China; 2 Shanghai Caoyang Community Health Care Center, Shanghai 200062, PR China; 3 Shanghai Huayang Community Health Care Center, Shanghai 200042, PR China

## Abstract

**Background:**

Over the past two decades, the prevalence of overweight or obesity has increased in China. The aims of this study were to firstly assess the baseline prevelences and the risk factors for overweight and obesity, and secondly to detect the changes of body mass index (BMI) over a follow-up period in Chinese adults in Shanghai.

**Methods:**

The data set of a population-based longitudinal study was analyzed. Anthropometric and biochemical data were collected for 5364 subjects (aged 25–95 years) during a period of 1998–2001. Among those individuals, 3032 subjects were interviewed and reexamined at the second survey from 2003 to 2004. Then the standardized prevalences for overweight and obesity were calculated using baseline data; the possible contributing factors of overweight and obesity were detected using binary logistic regression analysis; and the changes of BMI were evaluated after an average of 3.6-year follow-up period.

**Results:**

(1) According to the WHO standard and the Chinese standard, the sex- and age-standardized prevalences were 27.5% and 32.4% for overweight, and 3.7% and 9.1% for obesity, respectively. (2) The risks of overweight and obesity differed among different age groups. Family history of obesity increased the risk of overweight and obesity by about 1.2-fold for both genders. Current male smokers had a lower risk of overweight and obesity (OR = 0.76, *p *< 0.05) than nonsmokers. In contrast, current male drinkers had a higher risk of overweight and obesity (OR = 1.42, *p *< 0.05) than nondrinkers. Compared with low-educated women, medium- and high- educated women were at lower risk of overweight and obesity, and the corresponding ORs (95% CIs) were 0.64 (0.52–0.79) and 0.50(0.36–0.68), respectively. (3) The annual changes of BMI means ranged from an increase of 0.1 kg/m^2 ^to a decrease of 0.2 kg/m^2 ^(by genders and age groups). Meanwhile, the BMI increase was statistically significant in the 35–44 years age group, and the BMI decrease was significant above 65 years for both genders.

**Conclusion:**

This study showed high prevalence of overweight and obesity in Shanghai metropolis populations. The risk factors of overweight and obesity were multifactorial and gender specific. After 3.6 years, BMI means changed slightly, BMI increased mainly in middle-aged individuals and decreased in old individuals.

## Background

Obesity has long been recognized as one of the major health problems in many countries [1-3]. It is believed to be associated with increased morbidity and mortality, which are resulted from various chronic diseases, including cardiovascular disease, hypertension, type 2 diabetes, dyslipidaemia, and cancers [4-6].

The epidemic of obesity has been attributed to economic growth, rapid urbanization, and subsequent lifestyle changes [1,7]. The contribution of heredity factor may also partially account for obesity [8-10].

Over the past two decades, China has encountered a great economic development. As a result, the prevalence of overweight or obesity has significantly increased [11]. From 1992 to 1998, Shanghai (the largest city in China) had a rapid increase in overweight rates (men: 77%; women: 39%) and in obesity rates (men: 62% and women: 129%) according to the WHO BMI standard [12].

The aims of the present paper are to: (1) evaluate the prevalences of overweight and obesity; (2) assess the contributing factors associated with overweight and obesity; (3) detect the changes of BMI after 3.6 years in Shanghai metropolitan populations.

To our knowledge, systematic assessment of the prevalences, the risk factors and the trends for overweight and obesity using a population-based longitudinal study of Shanghai has seldom been reported.

## Methods

### Survey and study population

The present analysis was based on the data set of a community-based prospective study, i.e. Shanghai Diabetes Study (SHDS) [13]. The aims of the study were to assess the changes in the prevalence of metabolic syndrome and the association between the metabolic syndrome and sequent cardiovascular events over a follow-up period in two Shanghai communities.

The population of the study has been described elsewhere [13,14]. Shanghai had a population of about 16.4 million in 2000 with more than one hundred communities. Huayang and Caoyang were two medium-income urban communities in Shanghai and were selected for this study.

The cohort subjects were enrolled from the two urban communities with multistage sampling schemes (cluster, sex- and age-stratified, random). Pregnant women and those who were suffered from cancer, severe disability or severe psychiatric disturbance were excluded. At baseline, 2978 subjects were enrolled from September to November in 1998, April to September in 1999, and April to August in 2000 in Huayang community; and 3016 subjects were enrolled from July to November in 2001 in Caoyang community. The response rates were 97% in Huayang and 93% in Caoyang. A total of 5994 subjects aged from 15 to 95 years old were recruited and examined at baseline. Among these participants, 5364 subjects were aged from 25 to 95 years and were analyzed for this study.

Subsequently, a follow-up survey was conducted between December 2003 and February 2004 in Huayang community; between June 2004 and November 2004 in Caoyang community. Quite a few subjects were lost to follow-up later during an average 3.6-year follow-up. Finally a total of 3168 participants (aged ≥ 15 years at baseline) were interviewed and reexamined, among which 3032 subjects were aged 25–95 years (at baseline).

Informed consent was obtained from each participant. The protocol was in accordance with Helsinki Declaration, and was approved by the local ethical committee.

### Measurements

The 6^th ^hospital served as a coordinating and data center of the study. The two community hospitals formed the operating and monitoring units. The investigation stuff, from the three hospitals, consisted of diabetic specialists, physicians, epidemiologists and nurses.

#### Measurements at baseline

Participants arrived at the community hospitals at 6 to 7 AM following an overnight fast. After venous blood samples were collected, each participant received a 75-g OGTT, except for those with a history of diabetes mellitus (DM). Plasma glucose, serum insulin and serum lipid profiles were determined. In addition, some anthropometric indices were measured, including height, weight, waist circumference (WC) and hip circumference. Note that the height and weight were measured to the nearest 0.1 cm and 0.1 kg, respectively, while the participants were barefoot and in light clothing using Height & Weight Scale. Then baseline BMI was calculated as baseline weight divided by baseline height squared (kg/m^2^). And waist-to-hip ratio (WHR) was waist circumference divided by hip circumference. Body fat percent (BF %) was measured using the Body Fat Analyzer (TBF-401, TANITA, US). Using the standard right arm-cuff method, the patient's blood pressure was measured three times (with a one-minute rest interval) with a mercury sphygmomanometer after at least five-minutes rest, then the means of three measurements were adopted. The methods used in this paper can be found elsewhere [13,14].

#### Measurements at follow-up survey after an average of 3.6 years

At follow-up survey, such items as metabolic syndrome components, including weight and height, were examined in the same way as that of baseline. Unfortunately, some heights of the subjects were not measured at the follow-up survey. So the 3.6-year BMI was calculated using baseline height and weight after 3.6 years, which we believed is short enough for the height changes to be neglectable.

### Questionnaire

Trained stuff administered a standardized questionnaire to each individual through face-to-face interviews with him or her during check-up interval at baseline and follow-up survey. The questionnaires were about the demographic and lifestyle data of each subjects, including education, socio-economic factors, smoking habits, alcohol intake, medical history, and family history of disease.

#### (1) Assessment of household income and educational level at baseline

Monthly household income was classified into 4 levels: 1) <1,000 yuan RMB; 2) 1,100–2,000 yuan RMB; 3) 2,100–3,000 yuan RMB and 4) >3,000 yuan RMB.

The educational levels were categorized into three groups: 1) low education (illiteracy, primary and secondary education, less than 8–9 years schooling), 2) medium education (high school education, 9–12 years schooling) and 3) high education (college or university education, more than 14 years schooling).

#### (2) Assessment of smoking and alcohol intake behavior at baseline

We designed two questions for current cigarette smoking (the mean daily cigarette consumption in the past year and duration of smoking) and three questions related to quitting behaviors (duration of smoking cessation, the mean daily cigarette consumption and duration of smoking before smoking cessation). Then three groups of individuals (nonsmoker, current smoker and ex-smoker) were identified. Nonsmokers were defined as those who had never smoked, or smoked only occasionally; current smokers were those who smoked at least one cigarette per day lasting for at least one year; ex-smokers were those who had regularly smoked in the past, but had quit for at least half year.

As for the alcohol intake behavior, the individuals were questioned about their types, frequencies and quantity of alcohol intake in the past one year. The subjects were divided into three groups (nondrinker, current drinkers and ex-drinker) according to the frequency and quantity for the three types of alcohol beverages, which included Chinese distilled spirit, beer and rice wine (a type of Chinese alcoholic beverage brewed directly from grains such as rice, millet or wheat. Such liquors contain less than 20% alcohol). Nondrinkers were those who never or only occasionally drank; current drinkers were those who drank 6 g or more alcohol per day on average for at least one year; and ex-drinkers were those who had been a drinker, but quit for at least half a year before the baseline survey.

For current drinker, the consumption of the three alcohol beverages was recorded. The mean daily consumption of alcohol beverage was estimated and was expressed as grams of Chinese distilled spirit, rice wine or bottles of beer per day in the past year, and alcohol consumed per day was equal to the sum of alcohol consumed per day from Chinese distilled spirit, rice wine and beer. Crude estimations were adopted, i.e. 10 grams of alcohol consumed were roughly equal to 25 gram Chinese distilled spirit, 80 gram rice wine or 250 ml (half bottle) beer.

#### (3) Assessment of the family history of obesity at baseline

The questionnaires included the family history of obesity, which were about their biological mother, father, brothers, or sisters (first-degree relatives). Then the individuals were classified into two categories: 1) those who reported that no first-degree relatives had been obese; 2) those who reported that at least one first-degree relative had been obese.

### Diagnosis criteria

The WHO or the Chinese BMI cut-off points were used to define overweight and obesity.

(a) WHO standard (WHO, 2000)[1]:

Underweight: BMI < 18.5 kg/m^2^;

Normal weight: 18.5 kg/m^2 ^≤ BMI < 25 kg/m^2^;

Overweight: 25 kg/m^2 ^≤ BMI < 30 kg/m^2^;

Obesity: BMI ≥ 30 kg/m^2^;

(b) Chinese standard (2002) proposed by the Working Group on Obesity in China (WGOC) [15]

Underweight: BMI < 18.5 kg/m^2^;

Normal weight: 18.5 kg/m^2 ^≤ BMI < 24 kg/m^2^;

Overweight: 24 kg/m^2 ^≤ BMI < 28 kg/m^2^;

Obesity: BMI ≥ 28 kg/m^2^;

Hypertension: SBP ≥ 140 mmHg or DBP ≥ 90 mmHg or hypertension having been diagnosed and being received anti-hypertension therapy;

Diabetic Mellitus: FPG ≥ 7.0 mmol/L or 2 hPG ≥ 11.1 mmol/L or diabetic mellitus having been diagnosed and being received therapy;

The following cut-off values, proposed by the Third Report of the National Cholesterol Education Program [16], were applied: high TC (≥5.18 mmol/L), high LDL-C (≥3.37 mmol/L), high TG (≥1.70 mmol/L), low HDL-C (<1.04 mmol/L for males and <1.30 mmol/L for females).

### Statistical analyses

The statistical analyses were performed with the SPSS 15.0 for Windows. We analyzed the cross-sectional study data of the 5364 subjects (aged 25–95 years) at baseline and examined the BMI changes of the 3032 subjects (aged 25–95 years at baseline, among the 5364 subjects) with complete data on BMI after an average 3.6-year follow-up. All analyses were done separately for men and women. The level of significance was p < 0.05 for all analyses.

(1) Descriptive values are expressed as mean ± SD or proportions. Differences were assessed by paired t-test for changes of BMI means (self before-after control), by analysis of variance (ANOVA) for multiple mean comparisons and by chi-square test for proportions.

(2) According to the sex- and age- structure of the 2000 Census in China, the prevalence of overweight/obesity was standardized using the direct method.

(3) Unconditional binary logistic regression analyses were used to assess the association between the potential contributing factors and "overweight and obesity", where a binary variable (either overweight and obesity or normal weight) was used as dependent variable and the potential contributing factors were used as independent variables. The odds ratios (ORs) and the corresponding 95% confidence intervals (CIs) were obtained with forward stepwise regression.

## Results

### 1. The data presented the baseline characteristics of the 5364 subjects (aged ≥ 25 years) in three BMI classes for men and women [see Additional file 1]

Compared with the normal weight groups, the means of all variables were statistically higher in the overweight and obesity groups, except that the mean of HDL-C was lower for men and women; while those means were lower in the underweight groups, except for the means of HDL-C and age for both genders, and the means of LDL-C and FINS for women.

### 2. The data demonstrated the standardized prevalences of overweight/obesity [see Additional file 2]

According to the Chinese standard and the WHO standard, (1) the standardized prevalences of overweight plus obesity were 41.5% and 31.2%, respectively; (2) among women, the age-specific prevalences increased until age 74 for overweight but until age 64 for obesity, and then declined thereafter; among men, the age-specific prevalences peaked at 65–74 years old for overweight; (3) the age-specific prevalences of overweight were higher in men than in women of 25–54 years old, but the opposite was true for those above 55 years old; for obesity, the age-specific prevalences were higher in women than in men in all groups except for 25–34 years old.

The risk of overweight and obesity (BMI ≥ 25 kg/m^2^), reflected by adjusted ORs, increased from the age groups (25–34 years in women, and 35–44 years in men) to the 65–74 years age groups (in both genders), and then decreased.

### 3. The data summarized the risk factors of overweight and obesity for men and women in logistic regression models [see Additional file 3]

Among different categories of educational levels (for both genders), smoking behavior(for men), alcohol intake (for men) and household income (for women) the proportions of subjects were significantly different between the two BMI classes (p < 0.05). The proportions of the individuals with the family history of obesity were higher in the overweight and obesity group than in the normal weight group for both genders (p < 0.001).

The associations between the risk of overweight plus obesity and the following factors in multivariate models were as follows: (1) the risk increased by approximately 1.2-fold for both genders with family history of obesity. (2) Current male smokers had a lower risk of overweight and obesity (OR = 0.76, *p *< 0.05) than nonsmokers. In contrast, current male drinkers had a higher risk (OR = 1.42, *p *< 0.05) than nondrinkers. (3) Compared with low-educated women, medium- and high- educated women were at lower risk of overweight and obesity, and the corresponding ORs (95%CIs) were 0.64(0.52–0.79) and 0.50(0.36–0.68), respectively.

In addition, we also described some baseline characteristics of smoking and alcohol intake habits as follows.

Among male subjects, the proportions of overweight plus obesity were 30.2%, 34.5% and 36.1% in current smokers, ex-smokers and non-smokers, respectively. As to amount and duration of smoking in 1131 current smokers (1033 men and 98 women), the medians (interquartile range) were 15 cigarettes/day (10 cigarettes/day -20 cigarettes/day) and 20 years (15 years -30 years), respectively.

The frequency distribution of 631 current drinkers (580 men and 51 women) among different alcohol beverages was listed below: 1) Chinese distilled spirit (n = 123), 2) rice wine (n = 246), 3) beer (n = 152), 4) mixed Chinese distilled spirit and rice wine (n = 17), 5) mixed Chinese distilled spirit and beer (n = 21), 6) mixed rice wine and beer (n = 48), or 7) mixed Chinese distilled spirit, rice wine and beer (n = 24). For Chinese distilled spirit, rice wine and beer, the Medians (interquartile range) of consumption were 100 grams (50 g/day-150 g/day), 200 grams (100 g/day-250 g/day) and 1 bottle (1 bottle/day -1 bottle/day), respectively.

### 4. The data showed associations between metabolic diseases/disorders and BMI [see Additional file 4]

The proportions of DM, hypertension, high serum triglyceride, high serum LDL and low serum HDL among the BMI classes elevated with increasing BMI, this trend also could be observed through the corresponding age-adjusted ORs. Although the proportions of high serum total cholesterol in the overweight/obesity classes were higher than those in the normal/overweight classes, there were not significant differences of the proportions between the overweight group and the obesity group, as well as between the normal weight group and the underweight group.

### 5. Table 1 presented the changes of BMI after an average 3.6-year follow-up

**Table 1:** Changes of BMI for 1264 men and 1768 women after an average 3.6-year follow-up

Population*	N	follow-up (month)	BMI			BMI ≥ 25 kg/m^2^		
			mean ± SD			*n *(%)		
			
			Baseline	Follow-up	*p*	Baseline	Follow-up	*p*
Men								
25–34 y	54	36	24.5 ± 3.4	24.8 ± 3.4	0.091	23(42.6)	24(44.4)	0.903
35–44 y	241	44	23.5 ± 3.2	23.9 ± 3.2	0.000	73(30.3)	85(35.3)	0.407
45–54 y	267	47	23.8 ± 3.1	23.9 ± 3.0	0.332	92(34.5)	97(36.3)	0.755
55–64 y	186	45	24.1 ± 3.0	24.1 ± 2.9	0.957	70(37.6)	68(36.6)	0.884
65–74 y	315	43	24.4 ± 3.1	24.1 ± 3.3	0.016	136(43.2)	136(43.2)	1.000
75–95 y	201	40	23.2 ± 3.5	22.8 ± 3.6	0.001	62(30.8)	62(30.8)	1.000
Women								
25–34 y	51	35	22.1 ± 2.8	22.1 ± 2.7	0.755	9(17.6)	7(13.7)	0.642
35–44 y	421	44	23.4 ± 3.2	23.7 ± 3.2	0.000	119(28.3)	130(30.9)	0.540
45–54 y	443	47	24.1 ± 3.4	24.2 ± 3.4	0.329	168(37.9)	169(38.1)	1.000
55–64 y	247	48	25.1 ± 3.5	25.0 ± 3.6	0.209	122(49.4)	119(48.2)	0.874
65–74 y	386	45	25.0 ± 3.6	24.6 ± 3.6	0.000	196(50.8)	174(45.1)	0.347
75–94 y	220	42	24.3 ± 4.0	23.7 ± 4.2	0.000	97(44.1)	80(36.4)	0.280

*: age at baseline

Mean differences were assessed by the paired t-test.

Proportion differences were determined by the Chi-squared test.

(1) The means of baseline BMI were less than 25 kg/m^2 ^in all age groups except for the 55-year and the 65-year age groups in women. After an average of 3.6 years, the means of BMI were still less than 25 kg/m^2 ^in all groups except for the 55-year age group in women. (2) After 3.6 years, the increases of BMI were statistically significant in 35–44 years old, and the decreases (of BMI) were significant above 65 years for both genders. (3) In general, the annual changes of BMI means were insignificant, ranging from an increase of 0.1 kg/m^2 ^to a decrease of 0.1 kg/m^2 ^for men, and ranging from an increase of 0.1 kg/m^2 ^to a decrease of 0.2 kg/m^2 ^for women. (4) There were no significant changes in the percentages of overweight and obesity in the same-gender and same-age groups after 3.6 years.

### 6. Figure 1 depicted the percentages switching between two BMI groups after 3.6 years (by gender and age group)

**Figure 1:**
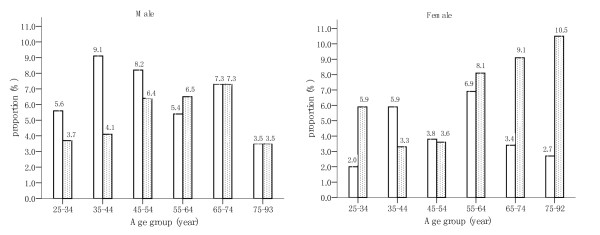
**The percentages switching into another BMI groups (by age group) for men and women after an average 3.6-year follow-up**. □ proportion switching from the underweight and normal weight group at baseline to the overweight and obesity group after an average 3.6-year follow-up. ■ proportion switching from the overweight and obesity group at baseline to the underweight and normal weight group after an average 3.6-year follow-up.

About 85%–93% of the subjects among different age groups remained in their original BMI groups after 3.6 years. At the same time, for young and middle-aged individuals (men aged 25–54 years and women aged 35–54 years), there were higher percentages switching from the underweight and normal weight group to the overweight and obesity group, than the opposite. But the case reversed for the old individuals (men aged 55–64 years and women above 55 years). The percentages transferring between the two BMI groups reached a balance for men above 65 years old.

## Discussion

Although BMI is not a perfect measure of body fat, it is practical in epidemiologic researches [17]. Considering that gender differences in the prevalences and the risk factors of overweight and obesity had been found in previous studies [18,19], all analyses were done separately for men and women in this paper.

### 1. The standardized prevalences

According to the WHO BMI cut-offs, the national adults prevalences of overweight and obesity were 12.9% and 1.5% in 1992 (the average prevalences were over 44485 subjects aged 20–60 years old) [20]; 18.28% and 2.48% in 1995.7–1997.6 (the prevalences were standardized by the structure of Chinese census 1990 over 42751 subjects aged 20–74 years old) [21]; as well as 18.9% and 2.9% in 2002 (the prevalences were standardized by the structure of Chinese census 2000 over 140022 subjects aged ≥ 18 years old) [22], respectively. In general, the prevalences of overweight and obesity are higher in northern areas than in southern areas, in big cities than in medium or small cities, in medium or small cities than in rural areas, and in developed areas than in developing areas [23].

In 2002, according to the Chinese BMI criteria and the structure of Chinese census 2000, the standardized prevalences of overweight for men and women were 31.1% and 25.8% in urban, and 19.6% and 21.4% in rural, respectively. In addition, 10.3% of the men and 9.5% of the women was obesity in urban, 4.9% of the men and 6.8% of the women was obesity in rural [22].

In our study, according to the Chinese BMI criteria, the standardized prevalences of overweight were 34.9% in men and 29.9% in women; the standardized prevalences of obesity were 8.3% in men and 10.0% in women. Our research showed an epidemic of overweight and obesity in Shanghai, the largest metropolitan city of China.

### 2. The risk factors of overweight and obesity for men and women

#### (1) Shared risk factors for both genders: age and family history of obesity

##### Age

Age has been considered as a prognostic factor of obesity for both genders in a series of published studies [18,19,24]. In this paper, the risk of overweight and obesity changed among the different age groups for men and women. So the age group variable (10-year age stratifications) was introduced as a covariate in the final models to erase the age confounding.

##### Family history of obesity

Family history of obesity is shown to be a main predictor for obesity [8,25,26]. It represents not only genetic susceptibility, shared environment, and common lifestyles, but also the interactions among them. Investigators had been advised to consider genetic factor in each study of human obesity. Our results in accordance with the previous studies [18,24], indicated about 1.2-fold increase in the risk of overweight and obesity for both genders with family history of obesity.

#### (2) Nonshared risk factors for both genders: smoking behaviors (in men), alcohol intake (in men) and education (in women)

##### Smoking behaviors

Lower risk of overweight and obesity was shown among current smokers than nonsmokers (OR = 0.76, *p *< 0.05) in men in this research. The negative association had also been found in some other cross-sectional studies [19,27-29]. A cohort study has also reported that longitudinal BMI increased less in smokers than in nonsmokers among middle-age subjects and decreased more in smokers than in nonsmokers among old-age subjects [30]. Although weight gain after quitting smoking was commonly cited in men [31,32], the correlation between smoking cessation and risk of overweight and obesity was not detected in our study.

##### Alcohol intake

The relation between alcohol intake and body weight was found to be either positive in the previous study [33]or nonexistent in other previous study [34]. This relation was affected by frequency, quantity and type of alcohol, and also affected by additional calories intake accompanied with alcohol intake [35-37]. In our study, most alcohol drinkers were light to moderate drinkers (see the characteristics of alcohol intake habits described in the result); and current male drinkers had a higher risk of overweight and obesity than nondrinkers (OR = 1.42, p = 0.002). Because few subjects were ex-drinkers, statistical difference was not detected between ex-drinkers and nondrinkers.

In China, smoking and alcohol drinking are much less common in women than in men. In our study, sex difference was significant for smoking and alcohol intake behaviors. Male smokers accounted for 91.3% of 1131 current smokers. And 91.9% of 631 current drinkers were male, which may partly explain the gender difference in the risk profiles.

##### Education

The inverse association had been found between education and obesity in both genders in previous studies[19,28,38], and had been found only in women in some other studies [39]. Our result appears to be supportive of the latter. Compared with low-educated women, medium- and high- educated women were at a lower risk of overweight and obesity.

##### Household income

Over the last few decades in the industrialized nations, a transition from a positive to a negative association had been seen between household income and obesity [40,41]. In this study, the negative association was found in women in univariate model but not found in multivariate model. One possible reason was that education and income were correlated with each other and education was more strongly associated with the risk of overweight and obesity than income in this population.

### 3. Obesity associated with metabolic diseases/disorders

Obese people commonly experience hypertension, lipidemia, and impaired glucose tolerance [1,5,6]. With increasing BMI, the proportion of diabetes, hypertension and dislipidemia gradually elevated. The dose-response relationships supported the possibility that a cause-and-effect relationship existed between increasing BMI and metabolic diseases/disorders, and that the underweight individuals had better health in metabolic status than those with normal weight in southern Chinese.

### 4. Changes of BMI after an average of 3.6 years

The means of BMI changed slightly over 3.6 years (see Table 1). Being consistent with the results of Table 1, Figure 1 suggested that the individuals were most likely (85%–93%) to remain in their original BMI groups among different age groups after 3.6 years. Meanwhile, for young and middle-aged individuals, there was a higher proportion switching from the underweight and normal weight group into the overweight and obesity group than that of reverse switching. But this case was not true for the old individuals. Some similar trends in BMI had been demonstrated in previous researches [42-44]. Therefore, for combating the current epidemic of obesity, more attention should be paid to the young and middle-aged individuals.

### 5. Limitation

The limitation of our study was that many subjects were lost during the follow-up period, and only 3168 of the baseline subjects were recalled at the second follow-up survey. The main reason for lose-up was that many subjects transferred to other communities as a result of the city reconstruction, which accounted for about 60% of all the lost subjects. Other reasons included noncooperation, being away from home, illness and death.

There were some statistical differences between the subjects followed up vs. those lost to follow up at the second visit. The differences included sex, age, education, income (in male), and drinking (in male). So we only detected changes of BMI for each individual using self before-after control analysis after an average 3.6 years in our paper.

## Conclusion

This study reports the high prevalence of overweight and obesity in Shanghai metropolis adults. The risk factors of overweight and obesity are multifactorial and gender specific. After an average of 3.6 years, most individuals remain in their original BMI groups. Meanwhile, the slight increase of BMI is found mainly in the middle-aged individuals.

These results support the notion that some risk factors can be used to identify individuals with high risk of overweight and obesity. These risk factors include family history of obesity, some behavior habits (alcohol intake) and education. Therefore, a community-based multiple strategies are urgently required to combat increasing prevalence of overweight and obesity in Shanghai communities.

## Competing interests

The authors declare that they have no competing interests.

## Authors' contributions

HL, YZ and HG participated in the data collection; WJ, YB, KX participated in the design of this study and general supervision of this research group; XH performed the statistical analysis and drafted the manuscript. SJ helped to check the data. All authors have read and approved the final manuscript.

## Pre-publication history

The pre-publication history for this paper can be accessed here:



## Supplementary Material

Additional file 1 **Baseline characteristics of the 5364 subjects classified by WHO BMI cut-offs.** The data presented the baseline characteristics of the 5364 subjects (aged ≥ 25 years) in three BMI classes for men and women. Statistical significances were determined with analysis of variance (ANOVA) for multiple mean comparisons. a*: p < 0.05, a**: p < 0.001 the underweight group (BMI < 18.5 kg/m^2^) vs. the normal weight group (18.5 kg/m^2 ^BMI ≤ 25 kg/m^2^); b*: p < 0.05, b**: p < 0.001 the overweight and obesity group (BMI ≥ 25 kg/m^2^) vs. the normal weight group (18.5 kg/m^2 ^≤ BMI < 25 kg/m^2^). Abbreviations: BMI: body mass index, WC: waist circumference, WHR: waist-to-hip ratio, WHtR: waist-to-height ratio, BF%: body fat percent, SBP: systolic blood pressure, DBP: diastolic blood pressure, TG: triglyceride, TC: total cholesterol, HDL-C: high density lipoprotein cholesterol, LDL-C: low density lipoprotein cholesterol, FPG: fasting plasma glucose, FINS: fasting insulin. Click here for file

Additional file 2 **Age- and sex-standardized means and proportions of overweight/obesity in the 5364 subjects at baseline.** The data demonstrated the standardized prevalences of overweight/obesity in the 5364 subjects (aged ≥ 25 years). a: The mean and proportion were standardized using the direct method according to the sex- and age-structure (aged ≥ 25 years) of the 2000 Census in China. OR: odds ratio; CI: confidence interval. Adjusted ORs reflected the associations between the age groups (10-year age groups) and overweight and obesity (BMI 25 ≥ kg/m ^2^). They were derived from multivariate logistic regression modesl with forward method. In male, the adjusted ORs were adjusted for family history of obesity, alcohol intake and smoking. In female, the adjusted ORs were adjusted for family history of obesity and education. Click here for file

Additional file 3 **Associations between the risk factors and overweight plus obesity in men and women.** The data summarized the risk factors of overweight and obesity for men and women in logistic regression models. Control: the subjects with 18.5 kg/m^2 ^≤ BMI < 25 kg/m^2^. Case: the subjects with BMI ≥ 25 kg/m^2^. OR: odds ratio; CI: confidence interval. Crude ORs were derived from univariate logistic regression analysis. Adjusted ORs were derived from multivariate logistic regression models with forward stepwise method. For men, the entered variables included age groups (the ORs were presented in Additional file 2), family history of obesity, alcohol intake and smoking; the unentered variables included household income and educational levels. For women, the entered variables included age groups (the ORs were presented in Additional file 2), family history of obesity and education; the unentered variables included household income, smoking and alcohol intake. a*: P < 0.05; a**: P < 0.001. Click here for file

Additional file 4 **Associations between metabolic diseases/disorders and BMI in 5364 subjects.** The data showed associations between metabolic diseases/disorders and BMI. OR: odds ratio; CI: confidence interval. Adjusted ORs were adjusted for the age groups (10-year age groups) using multivariate logistic regression analysis. a*: P < 0.05; a**: P < 0.001. Click here for file
