# Supplementary material for: Risk factors for overweight and obesity, and changes in body mass index of Chinese adults in Shanghai
Source: BMC Public Health. 2008 Nov 21;8:389. doi: 10.1186/1471-2458-8-389 (PMC2632663; doi:10.1186/1471-2458-8-389)
Supplement: Additional file 1 — Baseline characteristics of the 5364 subjects classified by WHO BMI cut-offs. The data presented the baseline characteristics of the 5364 subjects (aged ≥ 25 years) in three BMI classes for men and women. Statistical significances were determined with analysis of variance (ANOVA) for multiple mean comparisons. a*: p < 0.05, a**: p < 0.001 the underweight group (BMI < 18.5 kg/m2) vs. the normal weight group (18.5 kg/m2 BMI ≤ 25 kg/m2); b*: p < 0.05, b**: p < 0.001 the overweight and obesity group (BMI ≥ 25 kg/m2) vs. the normal weight group (18.5 kg/m2 ≤ BMI < 25 kg/m2). Abbreviations: BMI: body mass index, WC: waist circumference, WHR: waist-to-hip ratio, WHtR: waist-to-height ratio, BF%: body fat percent, SBP: systolic blood pressure, DBP: diastolic blood pressure, TG: triglyceride, TC: total cholesterol, HDL-C: high density lipoprotein cholesterol, LDL-C: low density lipoprotein cholesterol, FPG: fasting plasma glucose, FINS: fasting insulin. [file 1471-2458-8-389-S1.pdf]

Variables	Men (n=2292) mean±SD			Women (n=3072) mean±SD		
	BMI＜18.5kg/m² (n=128)	18.5kg/m²≤BMI＜25kg/m² (n=1411)	BMI≥25kg/m² (n=753)	BMI＜18.5kg/m² (n=148)	18.5kg/m²≤BMI＜25kg/m² (n=1791)	BMI≥25kg/m² (n=1133)
Age (year)	54.7±18.3	54.4±16.3	56.5±15.4 b*	53.6±19.8	51.2±15.1	58.0±14.6 b**
BMI (kg/m²)	17.4±1.0 a**	22.3±1.7	27.2±1.9 b**	17.4±1.0 a**	22.1±1.7	27.7±2.4 b**
WC (cm)	66.1±5.0 a**	79.2±6.7	92.0±6.7 b**	62.7±4.9 a**	73.4±6.6	87.4±7.9 b**
WHR	0.80±0.06 a**	0.87±0.06	0.94±0.06 b**	0.76±0.06 a**	0.82±0.07	0.89±0.07 b**
WHtR	0.39±0.03 a**	0.47±0.04	0.55±0.04 b**	0.40±0.04 a**	0.47±0.05	0.56±0.06 b**
BF%	13.2±3.7 a**	21.6±4.9	28.4±5.0 b**	19.86±3.2 a**	30.3.4±4.5	40.5±5.5 b**
SBP(mmHg)	117.3±16.5 a**	125.7±19.1	133.8±19.4 b**	117.1±20.9 a*	121.3±20.2	132.0±21.3 b**
DBP(mmHg)	73.7±7.7 a**	79.5±10.2	85.3±11.2 b**	73.0±9.4 a**	76.3±9.7	81.48±10.9 b**
TG(mmol/l)	1.17±0.58 a**	1.78±1.21	2.37±1.95 b**	1.29±0.59 a*	1.60±0.96	2.10±1.35 b**
TC(mmol/l)	4.46±0.94 a**	4.87±1.04	5.08±1.15 b**	4.98±1.29	5.00±1.16	5.33±1.19 b**
HDL-C (mmol/l)	1.34±0.27 a*	1.28±0.28	1.24±0.28 b*	1.45±0.27 a**	1.36±0.29	1.31±0.29 b**
LDL-C (mmol/l)	2.96±0.79 a**	3.35±0.91	3.55±0.99 b**	3.38±1.15	3.40±1.01	3.72±1.04 b**
FPG (mmol/l)	4.86±0.71 a*	5.23±1.53	5.61±1.56 b**	4.89±0.86 a*	5.16±1.31	5.64±1.51 b**
FINS (mU/l)	5.46±4.21 a**	7.21±4.39	10.63±6.14 b**	6.90±4.50	7.66±5.13	10.68±6.21 b**
