# Supplementary material for: Risk factors for overweight and obesity, and changes in body mass index of Chinese adults in Shanghai
Source: BMC Public Health. 2008 Nov 21;8:389. doi: 10.1186/1471-2458-8-389 (PMC2632663; doi:10.1186/1471-2458-8-389)
Supplement: Additional file 2 — Age- and sex-standardized means and proportions of overweight/obesity in the 5364 subjects at baseline. The data demonstrated the standardized prevalences of overweight/obesity in the 5364 subjects (aged ≥ 25 years). a: The mean and proportion were standardized using the direct method according to the sex- and age-structure (aged ≥ 25 years) of the 2000 Census in China. OR: odds ratio; CI: confidence interval. Adjusted ORs reflected the associations between the age groups (10-year age groups) and overweight and obesity (BMI 25 ≥ kg/m 2). They were derived from multivariate logistic regression modesl with forward method. In male, the adjusted ORs were adjusted for family history of obesity, alcohol intake and smoking. In female, the adjusted ORs were adjusted for family history of obesity and education. [file 1471-2458-8-389-S2.pdf]

Population	N	Chinese BMI cut-offs standard		WHO BMI cut-offs standard		
		24kg/m² ≤ BMI < 28kg/m²	BMI ≥ 28kg/m²	25kg/m² ≤ BMI < 30kg/m²	BMI ≥ 30kg/m²	BMI ≥ 25kg/m²
		Mean±SD (%)	Mean±SD (%)	Mean±SD (%)	Mean±SD (%)	adjusted OR(95%CI)
Men						
25-34y	215	25.8±1.1(32.6)	30.4±2.1(8.8)	26.9±1.3(27.4)	32.7±1.9(3.3)	1
35-44y	563	25.6±1.1(31.1)	29.9±1.7(6.0)	26.8±1.3(22.7)	31.5±1.8(2.3)	0.56(0.37-0.85)
45-54y	412	25.7±1.1(39.8)	29.6±1.4(8.7)	26.8±1.3(33.0)	31.4±1.1(2.4)	0.89(0.59-1.35)
55-64y	280	25.8±1.1(37.1)	30.2±1.5(8.9)	26.6±1.2(30.7)	31.5±0.8(4.6)	0.90(0.58-1.40)
65-74y	472	25.8±1.1(43.4)	29.8±1.7(10.6)	26.7±1.3(38.8)	31.9±1.4(3.4)	1.32(0.88-1.98)
75-95y	350	25.8±1.2(30.6)	29.4±1.6(8.9)	27.0±1.3(27.4)	32.0±2.1(1.7)	0.76(0.50-1.18)
Crude	2292	25.7±1.1(36.0)	29.8±1.7(8.5)	26.8±1.3(30.0)	31.7±1.5(2.8)	
Standardized [a]		25.7(34.9)	30.0(8.3)	26.8(28.6)	31.9(3.0)	
Women						
25-34y	226	25.5±1.1(23.5)	30.4±1.6(3.5)	26.5±1.3(16.8)	31.4±1.5(1.8)	1
35-44y	898	25.8±1.1(25.4)	30.1±2.2(8.5)	26.9±1.3(22.2)	31.9±2.3(3.6)	1.14(0.72-1.81)
45-54y	633	25.8±1.1(31.6)	29.8±1.9(12.8)	26.9±1.3(30.5)	31.6±2.1(4.9)	1.53(0.96-2.44)
55-64y	346	25.8±1.1(43.1)	30.8±2.3(18.5)	27.0±1.4(39.0)	32.4±2.1(9.8)	2.85(1.76-4.63)
65-74y	585	26.0±1.1(43.6)	30.6±2.6(17.6)	26.9±1.3(43.2)	32.7±2.4(8.4)	3.06(1.91-4.91)
75-94y	384	26.0±1.1(34.4)	30.3±2.1(16.7)	27.1±1.4(36.2)	32.2±1.9(6.8)	2.44(1.49-4.00)
Crude	3072	25.8±1.1(33.1)	30.3±2.3(12.9)	26.9±1.3(31.2)	32.2±2.2(5.7)	
Standardized [a]		25.7(29.9)	30.3(10.0)	26.8(26.3)	31.8(4.5)	
Total subjects						
Crude	5364	25.8±1.1(34.3)	30.2±1.7(11.0)	26.9±1.3(30.7)	32.1±2.0(4.5)	
Standardized [a]		25.7(32.4)	30.1(9.1)	26.8(27.5)	31.9(3.7)	
