# Supplementary material for: Risk factors for overweight and obesity, and changes in body mass index of Chinese adults in Shanghai
Source: BMC Public Health. 2008 Nov 21;8:389. doi: 10.1186/1471-2458-8-389 (PMC2632663; doi:10.1186/1471-2458-8-389)
Supplement: Additional file 3 — Associations between the risk factors and overweight plus obesity in men and women. The data summarized the risk factors of overweight and obesity for men and women in logistic regression models. Control: the subjects with 18.5 kg/m2 ≤ BMI < 25 kg/m2. Case: the subjects with BMI ≥ 25 kg/m2. OR: odds ratio; CI: confidence interval. Crude ORs were derived from univariate logistic regression analysis. Adjusted ORs were derived from multivariate logistic regression models with forward stepwise method. For men, the entered variables included age groups (the ORs were presented in Additional file 2), family history of obesity, alcohol intake and smoking; the unentered variables included household income and educational levels. For women, the entered variables included age groups (the ORs were presented in Additional file 2), family history of obesity and education; the unentered variables included household income, smoking and alcohol intake. a*: P < 0.05; a**: P < 0.001. [file 1471-2458-8-389-S3.pdf]

Variables	Men (N=2164)				Women (N=2924)			
	Control (N=1411) *n* (%)	Case (N=753) *n* (%)	Crude OR (95% C.I.)	Adjusted OR (95% C.I.)	Control (N=1791) *n* (%)	Case (N=1133) *n* (%)	Crude OR (95% C.I.)	Adjusted OR (95% C.I.)
Monthly household income (RMB)								
<1,000 yuan	251(19.2)	128(18.2)	1(Ref.)		334(20.7)	260(24.1)	1(Ref.)	
1,100yuan-2,000 yuan	532(40.8)	291(41.3)	1.07 (0.82 - 1.38)		680(42.1)	484(44.9)	0.93 (0.76 -1.13)	
2,100yuan-3,000 yuan	289(22.1)	147(20.9)	1.00 (0.74 -1.33)		361(22.3)	213(19.7)	0.76 (0.60 - 0.97) [a*]	
>3,000 yuan	233(17.9)	139(19.7)	1.16 (0.86 - 1.57)		241(14.9)	122(11.3)	0.65 (0.50 - 0.85) [a*]	
Education								
Low	570(43.6)	349(49.7)	1(Ref.)		891(55.4)	786(73.1)	1(Ref.)	1(Ref.)
Medium	447(34.2)	206(29.3)	0.75 (0.61 - 0.93) [a*]		544(33.8)	224(20.8)	0.47 (0.39 - 0.56) [a**]	0.64(0.52-0.79) [a**]
High	289(22.1)	147(20.9)	0.83 (0.65 - 1.05)		173(10.8)	65(6.0)	0.43 (0.31 - 0.58) [a**]	0.50(0.36-0.68) [a**]
Smoking								
Nonsmoker	517(39.6)	316(44.9)	1(Ref.)	1(Ref.)	1558(96.8)	1028(95.4)	1(Ref.)	
Current smoker	656(50.2)	312(44.3)	0.78 (0.65 - 0.95) [a*]	0.76(0.61-0.95) [a*]	47(2.9)	42(3.9)	1.34 (0.88 - 2.05)	
Exsmoker	134(10.3)	76(10.8)	0.93 (0.68 - 1.27)	0.83(0.60-1.15)	5(0.3)	8(0.7)	2.40 (0.78 - 7.37)	
Alcohol intake								
Nondrinker	971(74.1)	491(69.6)	1(Ref.)	1(Ref.)	1583(98.0)	1061(98.3)	1(Ref.)	
Current drinker	336(25.6)	212(30.1)	1.25 (1.02 - 1.53) [a*]	1.42(1.14 -1.77) [a*]	28(1.7)	18(1.7)	0.95 (0.52 - 1.73)	
Exdrinker	3(0.2)	2(0.3)	1.31 (0.22 - 7.89)	1.67(0.27-10.47)	4(0.2)	0	0.00	
Family history of obesity								
No	1185(90.6)	585(83.2)	1(Ref.)	1(Ref.)	1443(89.5)	897(83.3)	1(Ref.)	1(Ref.)
Yes	123(9.4)	118(16.8)	1.95 (1.49 - 2.56) [a**]	2.20(1.66-2.92) [a**]	169(10.5)	180(16.7)	1.71 (1.36 - 2.14) [a**]	2.25(1.77-2.86) [a**]
