# Supplementary material for: Risk factors for overweight and obesity, and changes in body mass index of Chinese adults in Shanghai
Source: BMC Public Health. 2008 Nov 21;8:389. doi: 10.1186/1471-2458-8-389 (PMC2632663; doi:10.1186/1471-2458-8-389)
Supplement: Additional file 4 — Associations between metabolic diseases/disorders and BMI in 5364 subjects. The data showed associations between metabolic diseases/disorders and BMI. OR: odds ratio; CI: confidence interval. Adjusted ORs were adjusted for the age groups (10-year age groups) using multivariate logistic regression analysis. a*: P < 0.05; a**: P < 0.001. [file 1471-2458-8-389-S4.pdf]

Metabolic diseases/disorders	BMI＜18.5kg/m^2 (N=276)		18.5kg/m^2≤BMI＜25kg/m^2 (N=3202)		25kg/m^2≤BMI＜30kg/m^2 (N=1645)		BMI≥30kg/m^2 (N=241)	
	n (%)	adjusted OR (95% CI)	*n* (%)	OR (ref.)	*n* (%)	adjusted OR (95% CI)	*n* (%)	adjusted OR (95% CI)
DM	16(5.9)	0.54 (0.32-0.92) [a*]	276(8.7)	1	288(17.6)	1.93(1.61-2.31) [a**]	57(23.8)	2.84(2.03-3.96) [a**]
Hypertension	52(18.8)	0.34(0.24-0.48) [a**]	1039(32.4)	1	893(54.3)	2.19(1.92-2.50) [a**]	168(69.7)	4.45(3.29-6.03) [a**]
high TG	50(18.1)	0.34(0.25-0.47) [a**]	1229(38.4)	1	976(59.3)	2.27(2.00-2.56) [a**]	147(61.0)	2.41(1.84-3.16) [a**]
high TC	89(32.2)	0.77(0.59-1.02)	1150(35.9)	1	812(49.4)	1.56(1.37-1.76) [a**]	114(47.3)	1.39(1.07-1.82) [a*]
low HDL-C	51(18.5)	0.60(0.44-0.82) [a*]	887(27.7)	1	587(35.7)	1.55(1.36-1.76) [a**]	96(39.8)	1.87(1.42-2.45) [a**]
high LDL-C	98(36.0)	0.64(0.49-0.84) [a*]	1405(44.1)	1	931(56.8)	1.47(1.30-1.67) [a**]	140(58.1)	1.52(1.16-2.00) [a*]
